# The representation of semantic similarities between object concepts in the brain: a hypergraph-based model

**DOI:** 10.1186/1471-2202-15-S1-P84

**Published:** 2014-07-21

**Authors:** Kaoutar Skiker, Mounir Maouene

**Affiliations:** 1 LIST Laboratory, FST, Abdelmalek Essaadi’s University, Tangier, Morocco; 2 Department of computer science, ENSAT, Abdelmalek Essaadi’s University, Tangier, Morocco

## 

The understanding of how semantic similarities between object concepts are reflected in the brain is a fundamental issue in cognitive neuroscience [1,2].fMRI studies have provided evidence that similar objects are grouped or clustered together into categories along a number of semantic features including visual, functional, tactile, sound, smell, taste and action features[3,4].

Traditionally, the relation between brain regions have been represented as graphs, where nodes denote brain regions and edges denote functional or structural connections between them[5].Neuroimaging studies have implicated a number of these regions, known as modality-specific areas, as representing specific knowledge of objects[4].These knowledge includes a number of visual ,functional and encyclopedic features. These features are of a particular interest because they allow people to make comparison between objects. Like brain networks, semantic similarities between objects may be represented as graphs; in that case, nodes represent objects and links represent the presence of a common feature. However, it is not possible to retrieve what constitute the meanings of these links.

**Figure 1:**
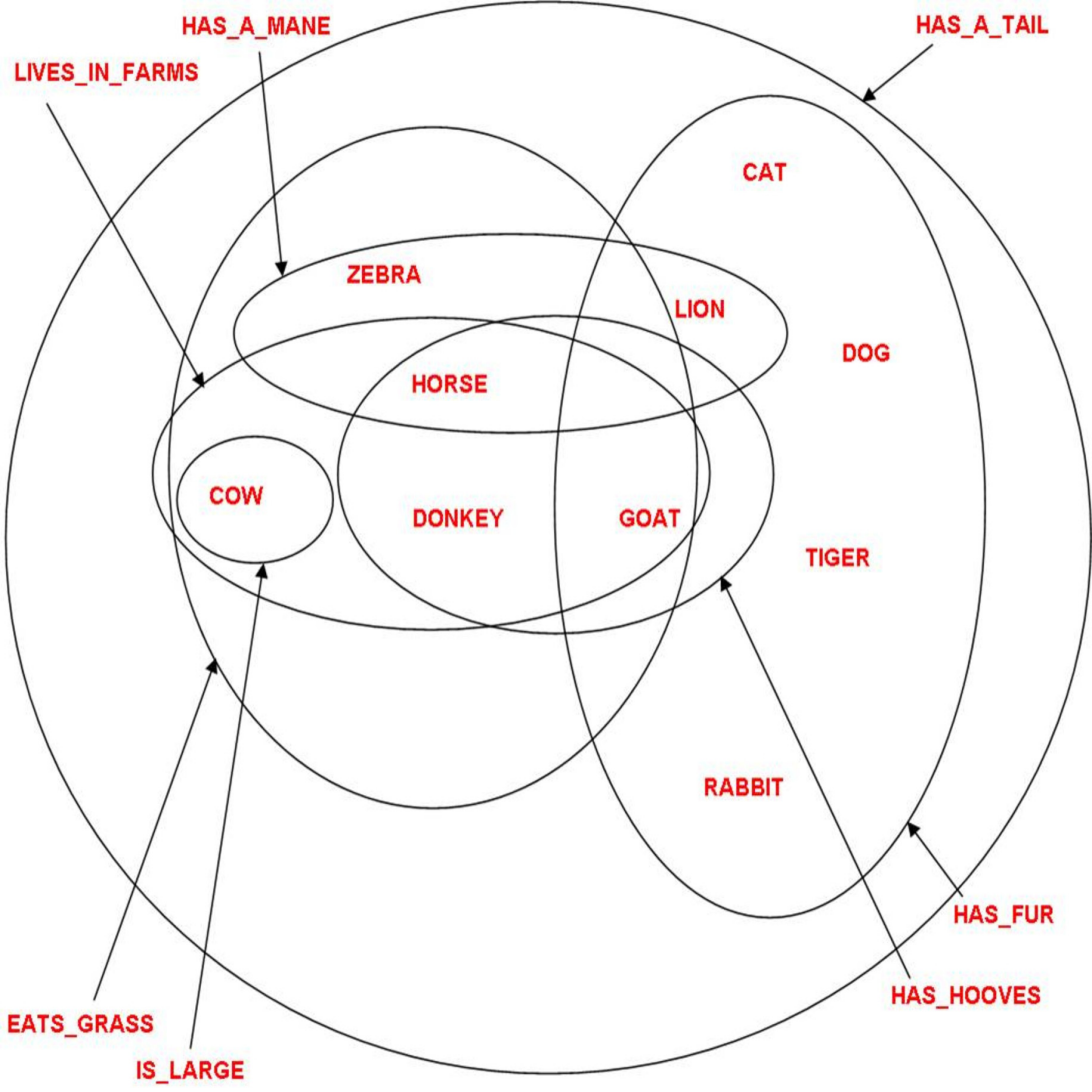
A hypergraph of the semantics similarities between animals

In this study, we propose to represent semantic similarities between objects with the mathematical model of hypergraph, a generalization of the graph model. The benefits of using hypergraphs for representing object similarities are threefold. First, links between objects, called hyperedges, connect a set of nodes and not just binary nodes as in graphs, such that the complexity of the similarities between objects can be preserved. Second, and unlike in graphs, links in hypergraphs keep their semantic, such that the semantic path between the similarities can be traced. Third, in a hypergraph, the clusters of objects overlap, because one category can share one or more of its semantic features with one or more categories and, by extension, a group of categories. We build a hypergraph for objects and their features based on the results available in neuroimaging studies including 1)-studies that have addressed the issue of how semantic similarities between object concepts are reflected in the brain [1,2], 2) studies that have examined how object concepts of different categories are organized in the brain [3,4,6], and also 3)-studies that have investigated the differences and similarities for processing object-related nouns and action-related verbs in the brain[7].

To summarize, hypergraphs provide an important approach for representing similarities between objects in the brain from the semantic perspective and provide a useful structure for representing clusters-based similarity.
